# A Note on Drawing Conclusions in the Study of Visual Search and the Use of Slopes in Particular

**DOI:** 10.1177/2041669516674220

**Published:** 2016-11-16

**Authors:** James T. Townsend

**Affiliations:** Indiana University, Bloomington, IN, USA

**Keywords:** current perspective, historical perspective, slope of set size, visual search

## Abstract

The slope of the set size function as a critical statistic first gained favor in the 1960s due in large part to the seminal papers on short-term memory search by Saul Sternberg and soon, many others. In the 1980s, the slope statistic reemerged in much the same role in visual search as Anne Treisman and again, soon many others brought that research topic into great prominence. This note offers the historical and current perspective of the present author, who has devoted a significant portion of his theoretical efforts to this and related topics over the past 50 years.

Within the now established and rather enormous field of visual search, Kristjansson (2015) argued forcefully in his original *i-Perception* article against the employment of slopes of set size functions. Wolfe (2016) responds that he agrees with many of the former author’s points but cautions against “throwing out the baby with the bath water,” due to this statistic’s overall utility. Kristjansson replies (this issue) that applying the Townsend and Ashby (1978) Inverse Efficiency Score to neutralize SAT effects, slope differences remain in his original experiment. This brief article is in response to the editor’s kind invitation to expand and reinterpret my original review in the form of a theoretical or philosophical or methodological note. Thus, the present note offers my perspective on these matters.

Although specific questions within this and similar issues can often be answered definitively by experimental facts, mathematics, or logic, many other questions lie on a continuum between “fact” and what must be judged as personal and debatable philosophy of science. I believe the following claims partake of some degree at several levels of debate.

- **Slopes from the Standpoint of the Architecture of Search** (usually restricted to parallel and serial architectures, with the understanding that our use of “architecture” does not necessarily imply immutability): I have maintained for almost 50 years that slopes, or more generally, increases in response times (RTs) as a function of set size, *n*, are primarily an indicant of work load capacity, not architecture. Thus, slopes generally serve as an ineffectual statistic to test architectures against one another. However, there is (and always has been) an asymmetry of logic here: Non-zero slopes are readily, and intuitively, produced by serial as well as limited capacity parallel models, but zero slopes or slopes associated with unlimited (or super!) capacity parallel models, are biologically and psychologically incompatible with serial processing.
- **Doctrines Concerning Slopes:** There are several assumptions associated with tying in the slope statistic with theories of search, as opposed to the slope performing simply as a descriptive statistic:
  1. Among these, perhaps most relevant to the current discussion and one emphasized by Kristjansson, is the principle that the slope should be an invariant across certain experimental manipulations such as response type. Such restriction is a valuable tool of theory construction—for instance, invariance is one of the most central concepts at all levels of modern physics. However, the scientist should always be aware of the extra theoretical baggage attending such an assumption. In the present milieu, this principle seems most compatible with a highly constricted version of serial processing. For instance, consider an experiment using Response Type A and suppose the data are well predicted by a standard serial model (i.e., the processing times are the same random variable for all items, are stochastically independent and additive). Now contemplate the parallel class of models that perfectly mimic this serial model. The invariant search axiom seems quite natural for the standard serial model when we move to experiments with Response Type B. It may seem far less cogent that parallel rates are such as to predict that invariance.
  2. With further regard to the theme just above, the conclusion that attentive visual search is serial has always been unwarranted or at least on shaky ground. The field of short-term memory search formerly made the same mistake of inferring that approximately straight line (and non-zero sloped) mean response time set size functions alone imply seriality (although it is important to mention that, unlike most others, the progenitor, Saul Sternberg (e.g., 1966), employed additional evidence such as addition of cumulant statistics, to back up his claims). Again stressing the asymmetric nature of inference here, flat mean RT set size pop out effects do falsify reasonable serial models. Additionally, it is not even clear that the huge corpus of memory set size curves in the literature are always straight lines, but rather better fit as log functions, as was emphatically demonstrated early on by Swanson & Briggs (1969). Recent evidence strongly points to early visual processing being unlimited capacity parallel with an exhaustive processing stopping rule which predicts a curve well approximated as a logarithmic function (Buetti, Cronin, Madison, Wang, & Lleras, 2016). If set size curves are not even straight lines, then much of the present-day inference-drawing based on slopes, seems ill advised. Finally, note that considerably more power in inference is bestowed when the scientist includes several stopping rules in the same study (e.g., see Townsend & Ashby, 1983, Chapter 4, Section: The Capacity Issue).
- **Nulling Out Speed Accuracy Tradeoffs:** Processing capacity has always been one of my major concerns from the very first papers on psychological processing systems (e.g., see Townsend, 1972, 1974). Of course, when accuracy varies, ever since the seminal works of psychologists like Wayne Wickelgren and Robert Pachella, we have realized that we must take into account both errors *and* speed when assessing capacity. Townsend and Ashby (1978) deliberate on many aspects of psychological processing systems relating to capacity, among them speed accuracy tradeoffs. They propose as a rough and approximate method of cancelling out speed accuracy tradeoffs, the statistic (employing Kristjansson’s terminology) inverse efficiencies (IES) = Mean RT/(1−Mean Error Rate). If the scientist knows the true model (impossible to be sure, and please observe the inescapable model dependency in this context), then the best way to null out speed accuracy tradeoffs is to estimate the parameter(s) associated with efficiency such as the serial or parallel rates of processing of, say, correct and incorrect information. IES will likely inevitably be a very coarse approximation to such a statistic. Although I (and I imagine Ashby) very much appreciate application of IES, more information would be helpful in proving that its use here justifies the inference concerning slope changes. For instance, if one can show (and this is potentially achievable) that IES is at least as conservative as, for instance, measuring the rates of processing in Poisson versions of serial or parallel models (see, e.g., Townsend & Ashby, 1983, Chapter 9), then the inference drawn by Kristjansson gains in credibility. Showing that a particular model provides excellent predictions and fits and then using the appropriate parameters to cancel out decisional effects would be even more impressive.

In conclusion, in my opinion, the science of visual search would profit by utilizing the now substantial battery of experimental procedures that provide quite precise and sturdy assessment of critical aspects of processing such as architecture, workload capacity, stopping rule, and independence. Many of these are distribution and parameter free. Then, such statistics such as slope and more generally the curvature of the mean RT set size function for various stopping rules can be engaged as dependent variables to study how various conditions, individual differences (including various pathologies), and experimental demands affect the underlying processing structure and mechanisms. In addition to a number of earlier treatments and surveys (available at http://www.indiana.edu/∼psymodel/), very up to date reviews are the following:

- Algom, D., Eidels, A., Hawkins, R. X. D., Jefferson, B., & Townsend, J. T. (2015). Features of response times: identification of cognitive mechanisms through mathematical modeling. In J. Busemeyer, J. Wang, A. Eidels, & J. T. Townsend (Eds.), *Handbook of computational and mathematical psychology* (1st Ed.). Oxford, England: Oxford University Press.
- Townsend, J. T., Wenger, M. J., & Houpt, J. W. (2016). Uncovering mental architecture and related mechanisms in elementary human perception, cognition and action. In E. J. Wagenmakers (Ed.), *The Stevens handbook of experimental psychology and cognitive neuroscience* (4th Ed.). Hoboken, NJ: Wiley
